# Antileishmanial Activity and Structure-Activity Relationship of Triazolic Compounds Derived from the Neolignans Grandisin, Veraguensin, and Machilin G

**DOI:** 10.3390/molecules21060802

**Published:** 2016-06-20

**Authors:** Eduarda C. Costa, Tatiana B. Cassamale, Diego B. Carvalho, Lauriane S. S. Bosquiroli, Mariáh Ojeda, Thalita V. Ximenes, Maria F. C. Matos, Mônica C. T. Kadri, Adriano C. M. Baroni, Carla C. P. Arruda

**Affiliations:** 1 Laboratório de Parasitologia Humana, Centro de Ciências Biológicas e da Saúde, Universidade Federal de Mato Grosso do Sul, 79090-900 Campo Grande—MS, Brazil; eduarda_c.costa@hotmail.com (E.C.C.); lauri.bosqui@gmail.com (L.S.S.B.); 2 Laboratório de Síntese e Química Medicinal-LASQUIM, Centro de Ciências Biológicas e da Saúde, Universidade Federal de Mato Grosso do Sul, 79090-900 Campo Grande—MS, Brazil; tatibortolo@gmail.com (T.B.C.); diegob.carvalho@hotmail.com (D.B.C.); adrianobaroni@hotmail.com (A.C.M.B.); 3 Laboratório de Biologia Molecular e Culturas Celulares, Centro de Ciências Biológicas e da Saúde, Universidade Federal de Mato Grosso do Sul, 79090-900 Campo Grande—MS, Brazil; mariahojeda@live.com (M.O.); matosmfc@gmail.com (M.F.C.M.); 4 Laboratório de Biofisiofarmacologia, Centro de Ciências Biológicas e da Saúde, Universidade Federal de Mato Grosso do Sul, 79090-900 Campo Grande—MS, Brazil; thalitario@hotmail.com (T.V.X.); monica.kadri@ufms.br (M.C.T.K.)

**Keywords:** neglected diseases, biological activity, cytotoxicity, synthetic compounds, neolignan derivatives

## Abstract

Sixteen 1,4-diaryl-1,2,3-triazole compounds **4**–**19** derived from the tetrahydrofuran neolignans veraguensin **1**, grandisin **2**, and machilin G **3** were tested against *Leishmania* (*Leishmania*) *amazonensis* intracellular amastigotes. Triazole compounds **4**–**19** were synthetized via Click Chemistry strategy by 1,3-dipolar cycloaddition between terminal acetylenes and aryl azides containing methoxy and methylenedioxy groups as substituents. Our results suggest that most derivatives were active against intracellular amastigotes, with IC_50_ values ranging from 4.4 to 32.7 µM. The index of molecular hydrophobicity (ClogP) ranged from 2.8 to 3.4, reflecting a lipophilicity/hydrosolubility rate suitable for transport across membranes, which may have resulted in the potent antileishmanial activity observed. Regarding structure-activity relationship (SAR), compounds **14** and **19**, containing a trimethoxy group, were the most active (IC_50_ values of 5.6 and 4.4 µM, respectively), with low cytotoxicity on mammalian cells (SI = 14.1 and 10.6). These compounds induced nitric oxide production by the host macrophage cells, which could be suggested as the mechanism involved in the intracellular killing of parasites. These results would be useful for the planning of new derivatives with higher antileishmanial activities.

## 1. Introduction

Cutaneous leishmaniasis (CL) is a parasitic infectious disease that affects the skin, cartilage and mucosa of the upper respiratory tract. The resulting ulcers can develop into destructive and disabling injuries, making the illness a serious public health problem [1]. There have been between 0.7 and 1.3 million new annual cases throughout the world, and 95% occur in the Americas, Mediterranean basin, Middle East and Central Asia [2]. *Leishmania (Leishmania) amazonensis* Lainson and Shaw 1972 is one of the etiological agents of CL. It produces the typical localized lesions or the diffuse form of the disease, in which the parasite spreads due to impaired cell-mediated immune response. Thus, the lesions tend not to heal spontaneously and to be more resistant to treatment [3,4].

The first choice drugs for CL are the pentavalent antimonials, which are associated to hepato, cardio and nephrotoxicity [5]. When these drugs are ineffective or cannot be prescribed, drugs such as amphotericin B, pentamidine or paramomycin are indicated, despite their degree of toxicity [6]. Resistance and high cost are other aspects that lead to the urgent need for new therapeutic options arising from natural products [7,8,9,10]. However, these products may have undesirable properties such as high toxicity, low solubility and bioavailability, which can be minimized by the development of synthetic derivatives [11].

Cassamale *et al.* [12] have synthetized a series of triazole derivatives from the tetrahydrofuran neolignans veraguensin **1**, grandisin **2**, and machilin G **3**. These compounds have been considered important scaffolds in molecular modification studies due to their antileishmanial and antichagasic activities [13,14,15,16]. Cassamale *et al.* [12] demonstrated the antileishmanial activity of these triazole derivatives on promastigote forms; now the present work shows their activity on *L. (L.) amazonensis* intracellular amastigotes, searching for structure-activity relationship information to support the development of new drug candidates for CL.

## 2. Results and Discussion

Synthetic derivatives of tetrahydrofuran neolignans **1**–**3** were classified as active (<20 µM), moderately active (20–50 µM), and potentially inactive (>50 µM) according to Upegui *et al.* [17]. Most compounds were considered active against *L.* (*L.*) *amazonensis* intracellular amastigotes (**4**, **6**, **8**, **9**, **10**, **11**, **12**, **14**, and **19**). Compounds **5**, **7**, **15**, **16**, **17**, and **18** have been moderately active and **13** did not show potential activity on the parasites (Table 1).

The ability to inhibit the growth of parasites apparently depends on the presence and ratio of lipophilic/hydrophilic substituents on aromatic rings [18]. The transport of a compound across membranes may be influenced by the molecular hydrophobicity described by the octanol/water partition coefficient (ClogP) [19]. According to Lipinski’s Rule of Five [20,21,22], compounds with logP < 5 have better absorption and permeation *in vivo*. Daunes and D’Silva [23] verified that among a series of molecules, the ones with higher logP (>2.7) were the most active against trypanosomatids once entering the host cell more easily. In other words, the molecular hydrophobic character improves the antileishmanial activity, which may indicate that the active compound must interact with a target system such as an enzyme or receptor, where the binding site is generally hydrophobic.

In our study, synthetic derivatives showed lipophilicity 2.8 < ClogP < 3.4 (Table 1), reflecting an adequate lipophilicity for the transport across membranes and resulting in potent antileishmanial activity.

We observed that ring B containing the trimethoxy substituent has influenced the antileishmanial activity independently of the substituent on the ring A (compounds **6**, **10**, **14** and **19**) (Table 1).

Grandisin derivative **6** was active on intracellular amastigotes (IC_50_ value of 9.4 µM), with the highest selectivity index. This compound was 66 times more toxic to amastigotes than to mammalian cells (Table 1). The substitution of tetrahydrofuran ring by triazolic ring may have resulted in increased activity, once it was described an IC_50_ value of 98.05 µM (42.4 µg·mL^−1^) for grandisin on *L. (L.) amazonensis* promastigotes [24]. Furthermore, its coefficient of hydrophobicity is lower than of its precursor **2** (2.8 and 3.7, respectively), which may have resulted in a better solubility and consequent *in vitro* activity. Indeed, more soluble grandisin derivatives have been synthetized in order to reduce its lipophilicity, which may limit *in vivo* studies [25]. It is important to note that the intracellular amastigote form is the target for drug candidates in the mammalian host.

**Figure 1:**
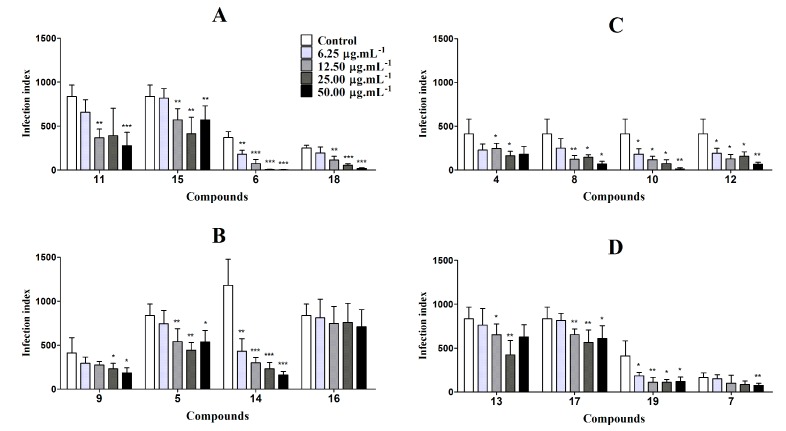
Effect of 1,4-diaryl-1,2,3-triazole derivatives (**4**–**19**) of the neolignans **1**–**3** on intracellular amastigotes. Peritoneal macrophages were infected with *L. amazonensis* and treated with different concentrations of the compounds. Infection index was calculated 24 h after treatment. Bars represent the mean ± SD of six replicates. * *p* ≤ 0.05, ** *p* ≤ 0.01, *** *p* ≤ 0.001 for the different concentrations *versus* untreated cells (control) (Student’s *t-*test). (**A**) trimethoxy series; (**B**) dimethoxy series; (**C**) methoxy series; (**D**) methylenedioxy series (substitution pattern of ring A).

**Figure 2:**
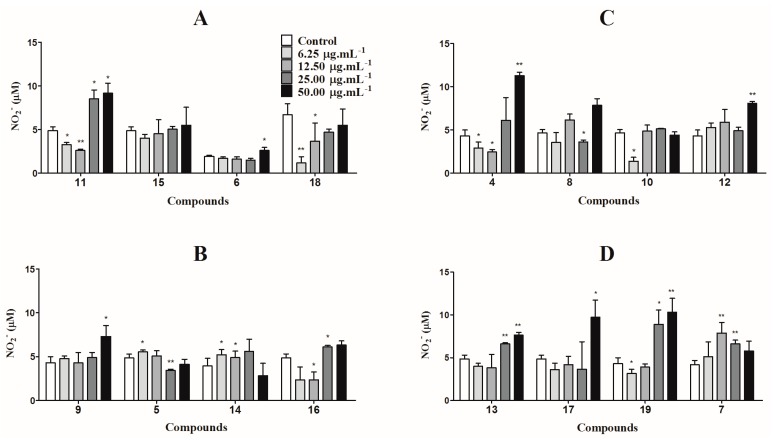
Nitric oxide release by *L. amazonensis* infected macrophages 24 h after treatment with 1,4-diaryl-1,2,3-triazole derivatives (**4**–**19**) of the neolignans **1**–**3**. Bars represent the mean ± SD of six replicates. * *p* ≤ 0.05, ** *p* ≤ 0.01, for the different concentrations compared to untreated cells (control) (Student’s *t*-test). (**A**) trimethoxy series; (**B**) dimethoxy series; (**C**) methoxy series; (**D**) methylenedioxy series (substitution pattern of ring A).

Treatment with compound **6** had significantly decreased the infection index at the concentration of 6.25 µg·mL^−1^, reaching 98.6% reduction (*p* < 0.0001) at the concentration of 50 µg·mL^−1^ (Figure 1A). **6** has induced an increased production of nitric oxide (NO) compared to untreated infected cells (control) (Figure 2A). Thus, NO production may be suggested as the mechanism of leishmanicidal action, especially because **6** does not seem to have a direct action on *L. (L.) amazonensis* once it was proven inactive on promastigote forms [12].

The other compounds from trimethoxy series (with substituents on ring A) were active (**11**) and moderately active (**15** and **18**), but less selective, with important cytotoxicity on mammalian cells (Table 1). All of them were able to significantly decrease infection index, mainly **18**, reaching 92.1% reduction (*p* < 0.0001) (Figure 1A). This compound, however, did not induce a significant production of NO. **11** acted variably on NO release, with a production lower than untreated cells at the lowest concentrations, but higher than control at the highest concentrations tested (Figure 2A).

Compound **5** with a dimethoxy group as substitution pattern on rings A and B (veraguensin **1** derivative) was moderately active on intracellular amastigotes (IC_50_ value of 21.3 µM) with relative selectivity (SI = 3.6). Silva Filho *et al.* [16] have demonstrated the activity of veraguensin on *L. donovani* promastigote forms (48.3 µM; 18 µg·mL^−1^). Once again we may educe that the insertion of triazole ring may have improved the antileishmanial activity due to reduction of octanol/water partition coefficient (3.1 (**5**) *versus* 4.2 (**1**)). Compound **5** has significantly reduced the infection index from the concentration of 12.5 μg·mL^−1^ (Figure 1B). Furthermore, the mechanism of leishmanicidal action seems also to be independent of NO, once NO production was lower than control at the concentration of 25 μg·mL^−1^ (Figure 2B). These data corroborate those obtained by Konishi *et al.* [26], who verified the inhibition of NO production from LPS-activated murine macrophages by three veraguensin’s position isomers.

The other compounds from dimethoxy series were active (**9** and **14**), excepting **16**, which was moderately active but selective (IC_50_ = 32.7 µM; SI > 23.5). Despite its moderate activity, **16** was able to significantly induce NO production (Figure 2B).

Compound **14** was active and selective (IC_50_ = 5.6 µM; SI = 14.1) (Table 1), and reduced the infection index at all concentrations tested (86.4% at the highest concentration, *p* = 0.0003) (Figure 1B). It’s important to note that **14** was a hybrid from veraguensin **1** and grandisin **2**, and it had the best activity of the whole dimethoxy series. Furthermore, it significantly induced NO production at the lowest concentrations tested, and this may be suggested as a possible mechanism of leishmanicidal action (Figure 2B).

Compounds from methoxy series showed IC_50_ values from 13.1 to 16.8 μM (**4**, **8**, **10**, and **12**, Table 1). Compound **4** was the most active of this series, and highly selective (IC_50_ = 13.1 µM; SI = 66.9). As well as **12**, this compound was able to induce an increase of NO production at the highest concentration tested (Figure 2C).

The most active compound on intracellular amastigotes (**19**) came from methylenedioxy series (IC_50_ = 4.4 µM). Compound **19** is a hybrid from machilin G **3** and grandisin **2** and was also quite selective (SI = 10.6) (Table 1). **19** has significantly reduced the infection index and in addition it induced NO production twice as high than control at the highest concentrations tested (Figure 2D). Cassamale *et al.* [12] have demonstrated this compound as highly active against *L.* (*L.*) *amazonensis* promastigote forms (IC_50_ = 7.2 µM), and this suggests its direct action on the parasite. On the other hand, position isomer **18** (IC_50_ = 29.8 μM, Table 1) was less active than **19**, indicating the role of minor structural differences on the antileishmanial activity of these compounds.

Compound **13** did not show potential activity and selectivity (Table 1). Despite this, **13** was able to induce an increase in NO production (Figure 2D), as well as its position isomer **12** (Figure 2C). We should point out that all compounds from methylenedioxy series have induced NO production in greater or lesser degree (Figure 2D). This implies that this group may be associated to cellular activation and/or cytotoxicity. For example, compound **17** (machilin G **3** analog) was toxic for the macrophages (SI = 0.3) with moderate antileishmanial activity (Table 1).

## 3. Materials and Methods

### 3.1. Triazole Derivatives of Neolignans

**Figure 3:**
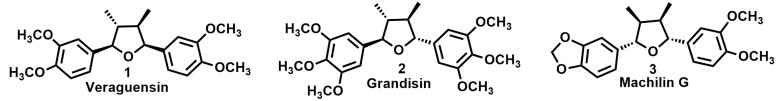
Structure of the neolignans veraguensin **1**, grandisin **2,** and machilin G **3**.

**Figure 4:**
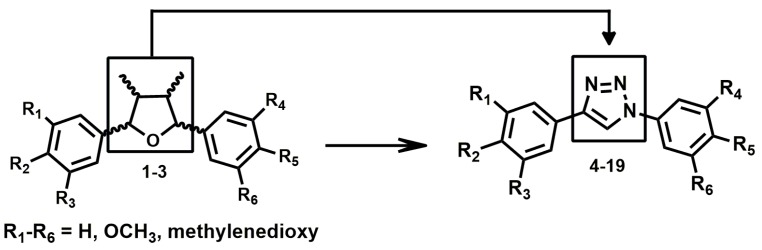
Structural design of 1,4-diaryl-1,2,3-triazole derivatives of the neolignans **1**–**3**.

Sixteen 1,4-diaryl-1,2,3-triazole derivatives with substitution patterns found in the neolignans veraguensin 1, grandisin 2, and machilin G 3 (Figure 3) were tested. They were designed based on the concept of the bioisosterism of rings, where the tetrahydrofuran core was substituted by a 1,2,3-triazole ring (Figure 4) [12].

Triazole derivatives **4**–**19** were obtained via Click Chemistry strategy from 1,3-dipolar cycloaddition reactions between terminal acetylenes **25a**–**d** and aromatic azides **27a**–**d** with methoxy and methylenedioxy substitution patterns [12]. The synthesis of starting materials began by preparing aryl bromides **21a**–**c** via a bromination reaction of **20a**–**c** in the presence of NBS, *p*-TsOH, CH_2_Cl_2_ and SiO_2_ [12,27]. Subsequently, a cross-coupling Sonogashira reaction between bromobenzenes **21a**–**c** and 2-methyl-3-butyn-2-ol in the presence of PdCl_2_(PPh_3_)_2_/CuI, Et_3_N provided the acetylene alcohols **22a**–**c** with 81%–86% yields, after 24 h reaction time [12,28].

**Scheme 1:**
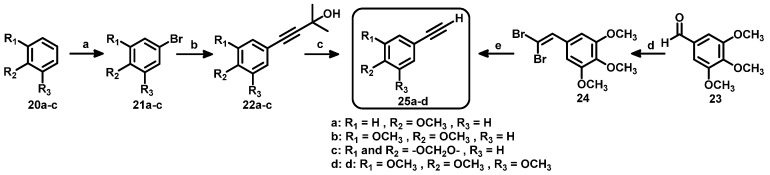
Synthesis of aryl acetylenes **25a**–**d** [12]. *Reagents and reaction*: (a) **20a**–**c** (75 mmol), NBS (75 mmol), TsOH (10 mmol), SiO_2_ (37 g), CH_2_Cl_2_ (210 mL), room temperature, 3 h, **21a** = 79%, **21b** = 82%, **21c** = 85%; (b) **21a**–**c** (50 mmol), 2-methyl-3-butyn-2-ol (183 mmol), PdCl_2_(Ph_3_)_2_ (2.5 mmol), CuI (5 mmol), Et_3_N (250 mL), reflux, 24 h, **22a** = 81%, **22b** = 82%, **22c** = 86%; (c) **22a**–**c** (30 mmol), KOH (90 mmol), toluene (225 mL), reflux, 24 h, **25a** = 75%, **25b** = 78%, **25c** = 79%; (d) CCBr_4_ (100 mmol), PPh_3_ (200 mmol), CH_2_Cl_2_ (100 mL), 0 °C, then 23 (50 mmol), 0 °C-room temperature, 5 h, 24 = 87%; (**e**) 24 (10 mmol), THF (50 mL), *n*-BuLi 2.5 M in hexanes (22 mmol), −25 °C—room temperature, 1 h, **25d** = 83%.

Retro-Favorski reaction of **22a**–**c** with KOH under reflux in toluene generated the terminal acetylenes **25a**–**c** with 75% to 79% yield [12,28,29,30]. Ethynyl-1,2,3-trimethoxybenzene **25d** was synthesized by the Corey-Fuchs method (Scheme 1) [12,31].

**Scheme 2:**
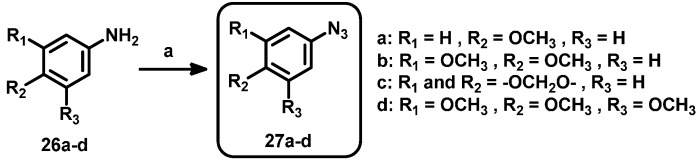
Synthesis of aryl azides **27a**–**d** [12]. *Reagents and reaction*: (a) **26a**–**d** (20 mmol), *t*-BuONO (43 mmol), CH_3_CN, 15 min, 0 °C, then TMSN_3_ (32.6 mmol), rt, 5–12 h; **27a** = 79%, **27b** = 86%, **27c** = 89%, **27d** = 90%.

Next, aromatic azides **27a**–**d** were prepared by the reaction of aromatic amines **26a**–**d** with *t*-BuONO/TMSN_3_ using the protocol reported by Moses *et al.* (Scheme 2) [12,32].

**Scheme 3:**
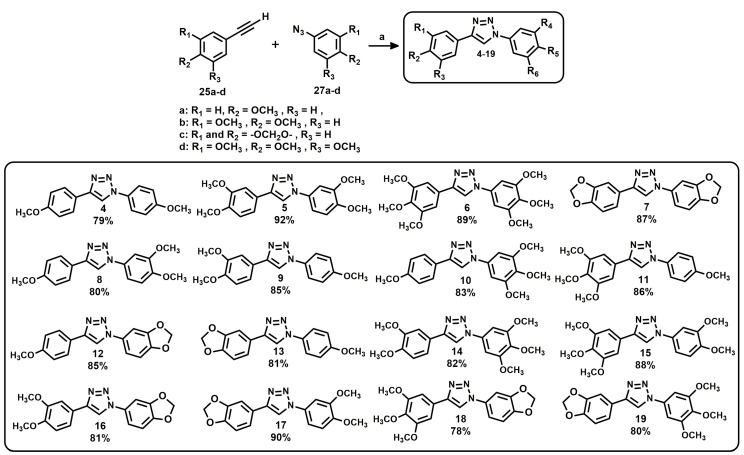
General method for the obtaining of 1,4-diaryl-1,2,3-triazole derivatives **4**–**19** [12,31]: (a) **25a**–**d** (2 mmol), **27a**–**d** (2 mmol), CuSO_4_·5H_2_O (0.128 mmol), sodium ascorbate (0.352 mmol), CH_2_Cl_2_/H_2_O (4 mL), room temperature, 24 h.

The 1,3-dipolar cycloaddition occurred when terminal acetylenes **25a**–**d** reacted with aryl azides **27a**–**d** using CuSO_4_·H_2_O, sodium ascorbate and CH_2_Cl_2_/H_2_O 1:1 as solvents, yielding the compounds **4**–**19** in 78% to 92% yield (Scheme 3) [12,33].

### 3.2. General Procedure for the Synthesis of Triazoles **4**–**19**

To a solution of terminal acetylenes **25a**–**d** (2 mmol, 1.0 equiv) and azides **27a**–**d** (2 mmol, 1.0 equiv) in dichloromethane (2 mL) and water (2 mL), were added CuSO_4_·5H_2_O (0.128 mmol, 0.064 equiv) and sodium ascorbate (0.352 mmol, 0.176 equiv). The reaction mixture was stirred for 24 h. Then it was added a saturated solution of NH_4_Cl (30 mL) and the product was extracted with dichloromethane (3 × 20 mL). The organic phase was dried over anhydrous MgSO_4_, and the solvent was evaporated under reduced pressure. The products were purified by recrystallization from ethyl acetate.

### 3.3. ClogP

In order to estimate the molecular hydrophobicity of triazole derivatives **4**–**19**, theoretical values of logP (ClogP) were calculated with ChemAxon software. The program estimated octanol/water partition coefficient based on a modified version of the method of Viswanadhan *et al.* [34].

### 3.4. Antileishmanial Activity

#### 3.4.1. Parasites and Peritoneal Macrophages

*In vitro* antileishmanial activity was evaluated on peritoneal macrophages infected with. *L.* (*L.*) *amazonensis* intracellular amastigotes. Parasites (IFLA/BR/1967/PH8 strain) were routinely isolated from BALB/c mice and maintained as promastigotes at 25 °C in *Schneider’s Insect Medium* (Sigma-Aldrich^®^, St. Louis, MO, USA) supplemented with 20% fetal calf serum (FCS-Cultilab^®^ Campinas, Brazil) and 140 µg·mL^−1^ gentamicin (Sigma-Aldrich^®^). Macrophages were obtained from peritoneal wash of BALB/c mice after euthanasia. 10 mL of RPMI 1640 (Sigma-Aldrich^®^) supplemented with 2% l-glutamine, 2.8% bicarbonate buffer, 100 U·mL^−1^ penicillin and 100 mg·mL^−1^ streptomycin were injected into the peritoneal cavity. After massage area, liquid was aspirated and transferred to tubes on ice. Peritoneal cells were quantified in Neubauer chamber after cellular exclusion with Trypan Blue staining (Sigma-Aldrich^®^).

#### 3.4.2. *In Vitro* Antileishmanial Activity on Intracellular Amastigotes

Peritoneal cells (1 × 10^5^ cells/well) were added to 24-well plates containing circular coverslips. Plates were incubated for one hour at 37 °C/5% CO_2_ to allow cell adhesion and then 1 × 10^6^ *L. (L.) amazonensis* promastigotes were added to each well. Plates were incubated at 35 °C/5% CO_2_ for four hours and then cells were treated for 24 h with synthetic compounds **4**–**19** (6.25–50 µg·mL^−1^). Amphotericin B (Sigma-Aldrich^®^) was used as the reference drug (0.25 to 2 µg·mL^−1^) and untreated cells were used as negative control. Coverslips were processed as described by Rizk *et al.* [35]. The overall number of amastigotes was determined by counting 100 cells in six replicates. The half maximal inhibitory concentration (IC_50_) was calculated using a nonlinear regression curve. Infection index was obtained as described by Paladi *et al.* [36].

### 3.5. Nitric Oxide Production

To evaluate the nitric oxide production (NO) by infected and treated peritoneal cells, 50 µL of culture supernatant from the antileishmanial assay were collected and incubated with equal volume of Griess reagent (1% sulfanilamide /0.1% naphthalene diamine in 5% phosphoric acid) for 10 min at room temperature. According to Ding *et al.* [37], the absorbance was determined at 540 nm and converted to NO_2_^−^ (µM) by comparing to a standard curve of known concentrations of sodium nitrite (1–10 µM) in RPMI medium (Sigma-Aldrich^®^).

### 3.6. Cytotoxicity Assay

Murine macrophages (J774.A1, Rio de Janeiro Cell Bank, Brazil) were seeded in 96-well plates (1 × 10^5^ mL^−1^) and incubated with compounds at 37 °C/5% CO_2_ for 48 h at concentrations of 0.25–250 µg·mL^−1^ to estimate IC_50_. Amphotericin B (Sigma-Aldrich^®^) was used as the reference drug (0.025–25 µg·mL^−1^). Cell growth was evaluated according to Skehan *et al.* [38] using the sulforhodamine B assay. Dimethyl sulfoxide (DMSO, Vetec^®^, Rio de Janeiro, Brazil) was used as negative control at the concentration used to solubilize the highest concentration of compounds. IC_50_ was calculated by nonlinear regression curve. Selectivity index was calculated according to Medeiros *et al.* [39].

### 3.7. Ethical Aspects

This study received approval from the local Animal Experimentation Ethical Committee (CEUA/UFMS) under protocol 503/2013.

## 4. Conclusions

Among 16 synthetic derivatives of tetrahydrofuran neolignans veraguensin **1**, grandisin **2** and machilin G **3**, 15 showed high or moderate antileishmanial activity. Compounds **14** and **19**, containing a trimethoxy substituent on ring B, were the most active against intracellular amastigotes, with low cytotoxicity on mammalian cells. These compounds induced nitric oxide production by the host macrophage cells, which could be suggested as the mechanism involved in the intracellular killing of parasites. These results would be useful for the planning of new derivatives with higher antileishmanial activities.

## Figures and Tables

**Table 1:**
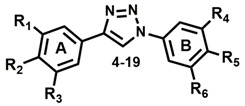
Molecular hydrophobicity, *in vitro* antileishmanial activity and cytotoxicity of 1,4-diaryl-1,2,3-triazole derivatives **4**–**19** of the neolignans **1**–**3**.

Compounds	R_1_	R_2_	R_3_	R_4_	R_5_	R_6_	ClogP ^1^	Intracellular Amastigotes IC_50_ (µM) ^2^	J774.A1 Cells IC_50_ (µM) ^3^	SI ^4^
**4**	-H	-OCH_3_	-H	-H	-OCH_3_	-H	3.4	13.1	877.2	66.9
**5**	-OCH_3_	-OCH_3_	-H	-OCH_3_	-OCH_3_	-H	3.1	21.3	76.4	3.6
**6**	-OCH_3_	-OCH_3_	-OCH_3_	-OCH_3_	-OCH_3_	-OCH_3_	2.8	9.4	>622.8	>66.2
**7**	-OCH_2_O-		-H	-OCH_2_O-		-H	2.9	20.4	86.2	4.2
**8**	-H	-OCH_3_	-H	-OCH_3_	-OCH_3_	-H	3.3	16.0	79.2	4.9
**9**	-OCH_3_	-OCH_3_	-H	-H	-OCH_3_	-H	3.3	18.3	22.6	1.2
**10**	-H	-OCH_3_	-H	-OCH_3_	-OCH_3_	-OCH_3_	3.1	16.8	65.4	3.9
**11**	-OCH_3_	-OCH_3_	-OCH_3_	-H	-OCH_3_	-H	3.1	16.5	67.5	4.1
**12**	-H	-OCH_3_	-H	-OCH_2_O-		-H	3.2	14.8	96.5	6.5
**13**	-OCH_2_O-		-H	-H	-OCH_3_	-H	3.2	50.7	134.6	2.6
**14**	-OCH_3_	-OCH_3_	-H	-OCH_3_	-OCH_3_	-OCH_3_	2.9	5.6	79.1	14.1
**15**	-OCH_3_	-OCH_3_	-OCH_3_	-OCH_3_	-OCH_3_	-H	2.9	29.2	76.9	2.6
**16**	-OCH_3_	-OCH_3_	-H	-OCH_2_O-		-H	3.0	32.7	>768.5	>23.5
**17**	-OCH_2_O-		-H	-OCH_3_	-OCH_3_	-H	3.0	29.9	9.2	0.3
**18**	-OCH_3_	-OCH_3_	-OCH_3_	-OCH_2_O-		-H	2.9	29.8	66.9	2.2
**19**	-OCH_2_O-		-H	-OCH_3_	-OCH_3_	-OCH_3_	2.9	4.4	46.9	10.6
Doxorubicin ^5^								-	0.5	-
Amphotericin B ^5^								0.7	2.2	3.1

^1^ ClogP, octanol/water partition coefficient; ^2^ IC_50_, half maximum inhibitory concentration on intracellular amastigotes; ^3^ IC_50_, half maximum inhibitory concentration on J774.A1 cells; ^4^ SI (selectivity index), IC_50_ on mammalian cells/IC_50_ on intracellular amastigotes; ^5^ Positive controls, amphotericin B for *L. (L.) amazonensis* and doxorubicin for J774.A1 cells. The data are representative of three independent experiments.

## Abbreviations

The following abbreviations are used in this manuscript:

C.L.	Cutaneous Leishmaniasis
FCS	Fetal Calf Serum
RPMI	Roswell Park Memorial Institute
IC_50_	Half maximal inhibitory concentration
DMSO	Dimethyl sulfoxide
S.I.	Selectivity Index

## References

[1] (2010). Brasil. Ministério da Saúde, Secretaria de Vigilância em Saúde. Manual de Vigilância da Leishmaniose Tegumentar Americana/Ministério da Saúde/ Secretaria de Vigilância em Saúde..

[2] WHO (World Health Organization) Leishmaniasis-Fact Sheet. http://www.who.int/mediacentre/factsheets/fs375/en/.

[3] Herwaldt B.L. (1999). Leishmaniasis. Lancet.

[4] Reithinger R., Dujardin J.-C., Louzir H., Pirmez C., Alexander B., Brooker S. (2007). Cutaneous leishmaniasis. Lancet Infect. Dis..

[5] Rath S., Trivelin L.A., Imbrunito T.R., Tomazela D.M., Jesús M.N., Marzal P.C. (2003). Antimoniais empregados no tratamento da leishmaniose: Estado da arte. Quim. Nova.

[6] Alviano D.S., Barreto A.L.S., Dias F.A., Rodrigues I.A., Rosa M.S.S., Alviano C.S., Soares R.M.A. (2012). Conventional therapy and promising plant-derived compounds against trypanosomatid parasites. Front. Microbiol..

[7] Croft S.L., Sundar S., Fairlamb A.H. (2006). Drug resistance in leishmaniases. Clin. Microbiol. Rev..

[8] Li J.W.H., Vederas J.C. (2009). Drug discovery and natural products: end of an era or an endless frontier?. Science.

[9] Lindoso J.A.L., Costa J.M.L., Queiroz I.T., Goto H. (2012). Review of the current treatments for leishmaniases. Res. Rep. Trop. Med..

[10] Newman D.J., Cragg G.M. (2007). Natural products as sources of new drugs over the last 25 years. J. Nat. Prod..

[11] Rishton G.M. (2008). Natural products as a robust source of new drugs and drug leads: Past successes and present day issues. Am. J. Cardiol..

[12] Cassamale T.B., Costa E.C., Carvalho D.B., Cassemiro N.S., Tomazela C.C., Marques M.C.S., Ojeda M., Matos M.F.C., Albuquerque S., Arruda C.C.P. (2016). Synthesis and Antitrypanosomastid Activity of 1,4-Diaryl-1,2,3-triazole Analogues of Neolignans Veraguensin, Grandisin and Machilin G. J. Braz. Chem. Soc..

[13] Lopes N.P., Chicaro R., Kato M.J., Albuquerque S., Yoshida M. (1998). Flavonoids and lignans from *Virola surinamensis* twigs and their *in vitro* activity against *Trypanosoma cruzi*. Planta Med..

[14] Oliveira R.B., Souza-Fagundes E.M., Siqueira H.A.J., Leite S.L., Donicci C.L., Zani C.L. (2006). Synthesis and evaluation of cytotoxic activity of arylfurans. Eur. J. Med. Chem..

[15] Oliveira R.B., Vaz A.B.M., Alves R.O., Liarte D.B., Donicci C.L., Romanha A.J., Zani C.L. (2006). Arylfurans as potential *Trypanosoma cruzi* trypanothione reductase inhibitors. Mem. Inst. Oswaldo Cruz.

[16] Silva Filho A.A., Costa E.S., Cunha W.R., Silva M.L., Nanayakkara D., Bastos J.K. (2008). *In vitro* antileishmanial and antimalarial activities of tetrahydrofuran lignans isolated from *Nectandra megapotamica* (Lauraceae). Phytother. Res..

[17] Upegui Y., Gil J.F., Quiñones W., Torres F., Escobar G., Robledo S.M., Echeverri F. (2014). Preparation of rotenone derivatives and *in vitro* analysis of their antimalarial, antileishmanial and selective cytotoxic activities. Molecules.

[18] Kayser O., Kiderlen A.F. (2001). *In vitro* leishmanicidal activity of naturally occurring chalcones. Phytother. Res..

[19] Nascimento J.P., Santos L.S., Santos R.H.A., Tozzo E., Ferreira J.G., Carmo M.C.L., Brasil D.S.B., Alves C.N. (2010). Synthesis, X-ray crystal structure and theoretical calculations of antileishmanial neolignan analogues. J. Braz. Chem. Soc..

[20] Lipinski C.A. (2000). Drug-like properties and the causes of poor solubility and poor permeability. J. Pharm. Toxicol. Meth..

[21] Lipinski C.A., Lombardo F., Dominy B.W., Feeney P.J. (2001). Experimental and computational approaches to estimate solubility and permeability in drug discovery and development settings. Adv. Drug Deliv. Rev..

[22] Veber D.F., Johnson S.R., Cheng H-Y., Smith B.R., Ward K.W., Kopple K.D. (2002). Molecular properties that influence the oral bioavailability of drug candidates. J. Med. Chem..

[23] Daunes S., D’Silva C. (2001). QSAR Study on the contribution of log P and Es to the *in vitro* antiprotozoal activity of glutathione derivatives. J. Med. Chem..

[24] Caldas J.P.A. (2010). Investigação da Atividade Antileishmania E Citotóxica das Neolignanas Burchelina, Grandisina E Licarina A. Master’s Thesis.

[25] Bernardes L.S.C., Kato M.J., Albuquerque S., Carvalho I. (2006). Synthesis and trypanocidal activity of 1,4-bis-(3,4,5-trimethoxy-phenyl)-1,4-butanediol and 1,4-bis-(3,4-dimethoxyphenyl)-1,4-butanediol. Bioorg. Med. Chem..

[26] Konishi T., Konoshima T., Daikonya A., Kitanaka S. (2005). Neolignans from *Piper futokadsura* and their inhibition of nitric oxide production. Chem. Pharm. Bull..

[27] Konishi H., Aritomi K., Okano T., Kiji J. (1989). A mild selective monobromination reagent system for alkoxybenzenes; *N*-Bromosuccinimide–Silica Gel. Bull. Chem. Soc. Jpn..

[28] Vuligonda V., Thacher S.M., Chandraratna R.A.S. (2001). Enantioselective synthesis of potent retinoid X receptor ligands: Differential biological activities of individual antipodes. J. Med. Chem..

[29] Dabdoub M.J., Baroni A.C.M., Lenardão E.J., Thiago R., Gianeti T.R., Hurtado G.R. (2001). Synthesis of (*Z*)-1-phenylseleno-1,4-diorganyl-1-buten-3-ynes: Hydroselenation of symmetrical and unsymmetrical 1,4-diorganyl-1,3-butadiynes. Tetrahedron.

[30] Dabdoub M.J., Dabdoub V.B., Lenardão E.J., Hurtado G.R., Barbosa S.L., Guerrero P.G., Nazário C.D., Viana L.H., Santana A.S., Baroni A.C.M. (2009). Synthesis of (*Z*)-1-Organylthiobut-1-en-3-ynes: Hydrothiolation of symmetrical and unsymmetrical buta-1,3-diynes. Synlett.

[31] Burroughs L., Ritchie J., Ngwenya M., Khan D., Lewis W., Woodward S. (2015). Anionic sigmatropic-electrocyclic-Chugaev cascades: Accessing 12-aryl-5-(methylthiocarbonylthio)tetracenes and a related anthra[2,3-b]thiophene. Beilstein J. Org. Chem..

[32] Barral K., Moorhouse A.D., Moses J.E. (2007). Efficient conversion of aromatic amines into azides: A one-pot synthesis of triazole linkages. Org. Lett..

[33] Lee B.Y., Park S.R., Jeon H.B., Kim K.S. (2006). A new solvent system for efficient synthesis of 1,2,3-triazoles. Tetrahedron Lett..

[34] Viswanadhan V.N., Ghose A.K., Revankar G.R., Robins R.K. (1989). Atomic physicochemical parameters for three dimensional structure directed quantitative structure-activity relationships. 4. Additional parameters for hydrophobic and dispersive interactions and their application for an automated superposition of certain naturally occurring nucleoside antibiotics. J. Chem. Inf. Comput. Sci..

[35] Rizk Y.S., Fischer A., Cunha M.C., Rodrigues P.O., Marques M.C.S., Matos M.F.C., Kadri M.C.T., Carollo C.A., Arruda C.C.P. (2014). *In vitro* activity of the hydroethanolic extract and biflavonoids isolated from *Selaginella sellowii* on *Leishmania (Leishmania) amazonensis*. Mem. Inst. Oswaldo Cruz.

[36] Paladi C.S., Pimentel I.A.S., Katz S., Cunha R.L.O.R., Judice W.A.S., Caires A.C.F., Barbieri C.L. (2012). *In vitro* and *in vivo* activity of a palladacycle complex on *Leishmania (Leishmania) amazonensis*. PLoS Negl. Trop. Dis..

[37] Ding A.H., Nathan C.F., Stuehr D.J. (1988). Release of reactive nitrogen intermediates and reactive oxygen intermediates from mouse peritoneal macrophages. Comparison of activating cytokines and evidence for independent production. J. Immunol..

[38] Skehan P., Storeng R., Scudiero D., Monks A., McMahon J., Vistica D., Warren J.T., Bokesch H., Kenney S., Boyd M.R. (1990). New colorimetric cytotoxicity assay for anticancer-drug screening. J. Natl. Cancer Inst..

[39] Medeiros M.G.F., Silva A.C., Citó A.M., Borges A.R., Lima S.G., Lopes J.A., Fiqueiredo R.C. (2011). *In vitro* antileishmanial activity and cytotoxicity of essential oil from *Lippiasidoides Cham*. Parasitol Int..

